# Oxidative Stress and Inflammatory Biomarkers in Male Infertility: A Narrative Review of Diagnostic Value and Clinical Integration

**DOI:** 10.3390/diagnostics16040527

**Published:** 2026-02-10

**Authors:** Athanasios Zikopoulos, Panagiotis Christopoulos, Theodoros Kalampokas, Angeliki Gerede, Efthalia Moustakli, Ioannis Arkoulis, Spyridon Topis, Anastasios Potiris, Chrysi Christodoulaki, Ioannis Tsakiridis, Themistoklis Dagklis, Sofoklis Stavros

**Affiliations:** 1 Torbay and South Devon NHS Foundation Trust Lowes Brg, Torquay TQ2 7AA, UK; thanzik92@gmail.com; 2 Second Department of Obstetrics and Gynecology, “Aretaieion” Hospital, Medical School, National and Kapodistrian University of Athens, 11528 Athens, Greece; panchrist@med.uoa.gr (P.C.); kalamp@yahoo.gr (T.K.); 3 Department of Obstetrics and Gynecology, Democritus University of Thrace, Alexandroupolis Campus, 69100 Alexandroupolis, Greece; agerede@otenet.gr; 4 Department of Nursing, School of Health Sciences, University of Ioannina, 45500 Ioannina, Greece; ef.moustakli@uoi.gr; 5 First Department of Obstetrics and Gynecology, Alexandra Hospital, Medical School, National and Kapodistrian University of Athens, 11528 Athens, Greece; garkoylis@hotmail.com (I.A.); spyros.topis1996@gmail.com (S.T.); 6 Department of Obstetrics and Gynecology, General Hospital of Chania, 73300 Chania, Greece; christodoulakichr@hotmail.com; 7 Third Department of Obstetrics and Gynecology, General Hospital Ippokratio, Medical School, Aristotle University of Thessaloniki, 54642 Thessaloniki, Greece; iotsakir@gmail.com (I.T.); tdagklis@gmail.com (T.D.)

**Keywords:** seminal plasma, redox imbalance, cytokine profiling, leukocytospermia, immune dysregulation, idiopathic infertility, biomarker-guided therapy

## Abstract

Conventional semen analysis frequently fails to identify the underlying pathophysiology of male infertility, which is a complicated clinical disease, especially in cases of idiopathic infertility. A growing body of research indicates that inflammation and oxidative stress (OS) are important and related factors in male reproductive failure. Excessive reactive oxygen species (ROS) promote lipid peroxidation, protein oxidation, mitochondrial dysfunction, and sperm DNA fragmentation, thereby compromising motility, morphology, and fertilizing capacity. Concurrently, pro-inflammatory mediators like interleukin-6 (IL-6), interleukin-8 (IL-8), and tumor necrosis factor-alpha (TNF-α) are frequently found in the seminal plasma of infertile men and are linked to poor semen parameters and testicular dysfunction. It is crucial that oxidative and inflammatory pathways work together to create a self-sustaining pathophysiological cycle that exacerbates sperm damage and destabilizes the reproductive milieu. The diagnostic significance, clinical suitability, and limitations of oxidative stress and inflammation biomarkers, such as malondialdehyde (MDA), 8-hydroxy-2′-deoxyguanosine (8-OHdG), total antioxidant capacity (TAC), and specific inflammatory markers, are critically assessed in this comprehensive review. The lack of established diagnostic thresholds, methodological variation, and translational issues that still restrict their widespread clinical implementation are highlighted in particular. Additionally, the potential contribution of biomarker-guided approaches to focused therapy decisions and individualized patient management is explored. This study examines how oxidative and inflammatory markers may complement conventional male infertility assessments by supporting more precise, mechanism-based approaches in reproductive medicine, while addressing diagnostic readiness and translational limitations.

## 1. Introduction

Infertility is a global health concern, affecting approximately 15% of couples of reproductive age, with male factors contributing to 40–50% of cases. Causes of male infertility are diverse and include congenital or acquired anatomical abnormalities, infections, systemic disorders, genetic defects, hormonal imbalances, and environmental or lifestyle factors [1,2]. Despite advances in assisted reproductive technologies (ART), a substantial proportion of male infertility cases remain classified as idiopathic, underscoring the need to elucidate the underlying genetic and cellular mechanisms [3].

The roles of inflammation and OS in male reproductive dysfunction have received increasing attention in recent decades. These interrelated mechanisms underly many clinical conditions that impact sperm function and production. OS arises when the balance between ROS generation and the antioxidant defense system is disrupted [4,5]. Although normal sperm maturation, capacitation, and the acrosome reaction depend on physiological levels of ROS, excessive ROS can cause lipid peroxidation, protein oxidation, and DNA breakage, which ultimately reduce sperm viability and fertilizing capacity [6].

Inflammation of the male reproductive tract has also been recognized as a significant contributor to infertility [7]. The sensitive testicular milieu that typically shields spermatogenesis is upset by inflammatory reactions, which are mediated by immune cells and cytokines [8]. Seminal plasma from infertile men often contains elevated levels of pro-inflammatory cytokines, including IL-6, IL-8, and TNF-α, which are frequently associated with abnormal semen characteristics. These elevated levels are often linked to aberrant semen characteristics. Conditions that can induce or exacerbate reproductive tract inflammation include infections, varicocele, systemic metabolic disorders, and autoimmune reactions [9].

Crucially, inflammation and OS act in a self-amplifying cycle rather than as isolated events. ROS can trigger pro-inflammatory pathways, while inflammatory mediators further exacerbate oxidative damage by recruiting leukocytes. This interaction highlights a shared pathophysiological mechanism that significantly contributes to male reproductive dysfunction [10,11].

The growing body of research has increased the importance of identifying reliable biomarkers of OS and inflammation with diagnostic and prognostic relevance in male infertility. The potential of biomarkers such as MDA, 8-OHdG, total antioxidant capacity (TAC), and newer composite tests to reflect sperm damage, redox balance, and inflammatory activity is increasingly being investigated [12,13]. The practical application of these biomarkers remains limited despite substantial research efforts, primarily due to the lack of established diagnostic thresholds, methodological heterogeneity, and uncertainty regarding their additional diagnostic value compared with traditional sperm analysis. However, treatment measures, like the use of antioxidant supplements, lifestyle changes, and anti-inflammatory techniques, have frequently been implemented without first classifying patients according to biomarkers [14].

This narrative review aims to objectively assess, from a diagnostic standpoint, the significance of inflammatory indicators and oxidative stress in male infertility. Beyond mechanistic pathways, this review evaluates the clinical relevance, diagnostic maturity, and limitations of available biomarkers, highlighting their potential role in the routine assessment of infertile men. This work aims to clarify how oxidative and inflammatory indicators can complement traditional testing and enable more individualized, mechanistically based methods in reproductive care by revealing translational gaps and unmet diagnostic needs.

## 2. Methodology

This narrative review critically synthesizes current evidence on oxidative stress and inflammatory indicators in male infertility, focusing on their diagnostic relevance and potential clinical application. Major electronic databases, including PubMed, Scopus, Web of Science, and Google Scholar, were systematically searched for relevant literature up to September 2025. To enhance the retrieval of pertinent literature, the search approach was based on a combination of Medical Subject Headings (MeSHs) and free-text phrases, including male infertility, oxidative stress, reactive oxygen species, sperm DNA damage, inflammation, cytokines, and biomarkers.

Original clinical trials, translational research, and related review articles that had undergone peer review and were published in English were given priority. To find more pertinent research, the reference lists of a few chosen publications were also manually reviewed. Studies that examined the relationship between male reproductive function or infertility outcomes and oxidative stress, inflammatory mediators, or associated biomarkers met the inclusion criteria. Conversely, studies conducted exclusively in animal models with limited translational relevance, conference abstracts lacking comprehensive data, single case reports, and non-English publications were excluded.

Given the focus of this narrative review on conceptual synthesis and diagnostic interpretation rather than quantitative data comparison, we did not perform a formal quality assessment or meta-analysis. The available literature on oxidative stress and inflammatory biomarkers in male infertility is characterized by substantial heterogeneity in study design, biomarker assays, laboratory methodologies, patient populations, and reported clinical outcomes, which limits the validity and clinical interpretability of pooled statistical analyses. To address potential sources of bias and variability, studies were selected based on relevance, translational value, and clinical applicability, and the evidence is discussed in a manner that distinguishes between biomarkers supported by more consistent clinical data and those that remain exploratory. These limitations are explicitly acknowledged to assist readers in contextualizing the strength and diagnostic maturity of the available evidence.

## 3. OS and Male Infertility

### 3.1. Sources of ROS

The male reproductive system continuously produces ROS, which play essential physiological roles in processes such as the acrosome reaction, sperm maturation, capacitation, and hyperactivation [15,16]. Sperm structure and function are disrupted by OS, which occurs when the production of ROS exceeds the capacity of antioxidant mechanisms to neutralize them. From a diagnostic perspective, understanding the origin and duration of ROS overproduction is critical, as it is reflected by measurable alterations in sperm redox balance [6].

Both endogenous and exogenous causes can lead to increased generation of ROS. Immature spermatozoa, which retain residual cytoplasmic droplets rich in glucose-6-phosphate dehydrogenase, are an important endogenous source of increased NADPH-dependent ROS generation [17,18,19]. Furthermore, sperm leukocytes, mainly neutrophils and macrophages, are a significant source of ROS, particularly when the male reproductive system is infected or inflamed [20], directly linking oxidative stress with inflammatory diagnostic indicators such as leukocytospermia.

Chronic oxidative imbalance is also maintained by lifestyle choices and environmental exposures, including alcohol consumption, smoking, obesity, an imbalanced diet, and exposure to environmental pollutants [6]. Clinically, these factors are often associated with increased oxidative stress biomarker levels in seminal plasma. Furthermore, several pathological conditions associated with male infertility, including varicocele, diabetes mellitus, metabolic syndrome, cryptorchidism, and testicular torsion, have been linked to elevated ROS levels [21]. This underscores the importance of focused evaluation of OS in specific patient populations.

### 3.2. Mechanisms of Oxidative Damage

Sperm are especially susceptible to damage from ROS due to their distinct structural features and low natural antioxidant defense [22]. Due to its high concentration of polyunsaturated fatty acids, the sperm plasma membrane is particularly vulnerable to lipid peroxidation. This process impairs motility, decreases membrane fluidity and integrity, and makes it more difficult for the sperm to attach to and fuse with the oocyte [19,23]. MDA, a byproduct of lipid peroxidation, serves as an indirect yet clinically relevant marker of oxidative damage to cell membranes.

ROS not only damage membranes but also oxidize proteins, changing the structure and function of cytoskeletal proteins, enzymes, and receptors that are essential for fertilization [24,25]. Oxidative stress causes single-stranded and double-stranded DNA breakage as well as oxidative base alterations at the genomic level, the primary one being the production of 8-OHdG [26,27]. These alterations accumulate and manifest as increased sperm DNA damage because spermatozoa are transcriptionally inactive and have a limited capacity for DNA repair. Clinically, increased oxidative DNA damage is associated with reduced fertilization rates, impaired embryo development, and RPL, underscoring its diagnostic relevance [28].

### 3.3. Clinical Correlates of OS

According to clinical research, OS is strongly correlated with abnormal sperm parameters. Oligozoospermia, asthenozoospermia, teratozoospermia, and increased sperm DNA fragmentation have all been associated with elevated levels of ROS in sperm [29,30,31]. These changes have a negative impact on implantation outcomes, fertilization rates, and embryo development, particularly in ART.

Moreover, oxidative stress reduces ATP synthesis and mitochondrial membrane potential, thereby impairing mitochondrial function and sperm motility [32]. Significantly, even in males with infertility that cannot be explained, elevated oxidative damage has been linked to poor reproductive results. Overall, these findings reinforce the role of oxidative stress as a key diagnostic target assessing idiopathic male infertility, demonstrating that it represents a primary pathophysiological factor rather than a secondary phenomenon [33]. A coherent framework for comprehending how oxidative stress translates into clinically detectable abnormalities is provided by Table 1, which lists the primary endogenous and exogenous sources of ROS, their methods of action, and the ensuing consequences on sperm structure and function.

## 4. Inflammation and Male Infertility

### 4.1. Role of Pro-Inflammatory Cytokines

The pathophysiology of male infertility is significantly influenced by inflammation in the male reproductive system [7,47]. Under normal circumstances, the immune system strikes a careful balance between providing effective protection against pathogenic pathogens and shielding sperm, which are cells with distinct antigenicity, from immune attack [48]. Spermatogenesis and sperm functioning can be negatively impacted by disruption of this balance, which is typified by excessive or prolonged production of inflammatory mediators [49]. From a diagnostic perspective, changes in the inflammatory profile of seminal plasma indicate this immunological impairment.

In fact, pro-inflammatory cytokines like IL-6, IL-8, TNF-α, and interferon–gamma (IFN-γ) are frequently found in higher amounts in the seminal plasma of infertile males [50]. These cytokines adversely affect reproductive function through several mechanisms, including impaired steroidogenesis in Leydig cells, disruption of Sertoli cell support of germ cells, and dysregulation of the immunoprivileged testicular environment. The oxidative–inflammatory vicious loop previously mentioned is further reinforced by cytokine-induced leukocyte recruitment and activation, which increases the generation of ROS [47,51].

Seminal plasma cytokines are primarily assessed in research settings; however, their consistent association with poor sperm quality supports their potential use as adjunctive diagnostic markers of inflammatory activity in specific clinical scenarios.

### 4.2. Clinical Associations of Inflammation

The strong correlation between inflammation and a variety of reproductive and systemic conditions highlights the clinical significance of inflammatory processes in male infertility [47]. Genital infections such as prostatitis, epididymitis, and orchitis trigger localized inflammatory responses that lead to leukocytospermia and increased seminal cytokine levels [20,52]. These changes adversely affect sperm motility, morphology, and DNA integrity.

Although leukocytospermia remains the most accessible clinical marker of inflammatory activity in semen, its diagnostic utility is limited by poor specificity and its inability to capture the complexity and spectrum of immune activation within the male reproductive tract. Importantly, the absence of leukocytospermia does not exclude biologically relevant inflammatory signaling, while its presence does not necessarily imply pathogenic inflammation. Consequently, leukocytospermia should be regarded as a screening marker rather than a definitive diagnostic criterion, ideally interpreted in conjunction with additional inflammatory mediators and oxidative stress markers [53,54]. In this context, the term “subclinical or low-grade inflammation” is used descriptively to denote measurable inflammatory signaling without overt infection or classical inflammatory findings, rather than to imply a uniformly pathogenic state.

Notably, even in the absence of clinically evident infections, males with unexplained infertility may exhibit impaired sperm quality, which has been associated in some studies with altered inflammatory signaling in the seminal environment [55]. Simultaneously, the pathophysiology of varicocele, one of the most prevalent and potentially surgically treatable causes of male infertility, has also been linked to inflammatory mechanisms. Increased expression of inflammatory mediators within the spermatic venous network causes oxidative damage and testicular dysfunction [56].

Moreover, persistent low-grade inflammation has been associated with impaired metabolic health and reduced male reproductive function in systemic disorders such as obesity, metabolic syndrome, and diabetes mellitus [57]. Lastly, a third inflammatory mechanism with important diagnostic and prognostic implications is autoimmune responses to sperm antigens, which can be brought on by trauma, infection, or surgical disruption of the testes’ protective barriers [58,59].

### 4.3. Immune Privilege and Disruption

The blood–testis barrier, the local generation of immunosuppressive cytokines, and the unique interactions between Sertoli cells and developing germ cells all contribute to the testes’ traditional status as an immune-privileged organ [60,61]. Since many sperm antigens are only expressed once central immunological tolerance has been established, this immune privilege is crucial. Therefore, spermatogenesis may be seriously impacted if inflammatory activities alter this protective microenvironment [62].

Sperm antigens may be exposed to the systemic immune system when the blood–testis barrier is compromised, as occurs during trauma, infection, or surgery, resulting in the generation of antibodies against sperm [63]. By decreasing sperm motility, obstructing sperm–oocyte contact, and interfering with capacitation, these antisperm antibodies can have a detrimental effect on fertility. Persistent inflammatory signaling can progressively weaken the immunological privilege of the testes even in the absence of clinically evident infections, resulting in chronic testicular dysfunction and ultimately infertility [64,65,66].

From a diagnostic standpoint, these immunologically induced changes emphasize how crucial it is to take immunological and inflammatory factors into account when assessing cases of male infertility that are otherwise unexplained [59,64,67]. In this regard, Table 2 provides a logical framework for comprehending how inflammatory activity translates into clinically noticeable reproductive dysfunction by summarizing the major inflammatory mediators implicated in male infertility, their primary sources, and their documented effects on spermatogenesis and sperm quality.

## 5. Crosstalk Between OS and Inflammation

### 5.1. Mutual Amplification of Pathways

The intimate and reciprocal relationship between OS and inflammation, which work as linked rather than separate biological processes, reinforces male reproductive failure. Pro-inflammatory cytokines, including TNF-α, IL-6, and IL-8, are promoted by redox-sensitive transcription factors, primarily nuclear factor kappa B (NF-κB), which are activated by ROS [76,77]. Further increases in ROS generation result from these cytokines’ facilitation of leukocyte recruitment and activation within the male reproductive tract. A self-sustaining oxidative–inflammatory cycle is produced by this mutually reinforcing signaling, which gradually impairs sperm structure and function [78,79].

This reciprocal interaction underscores a key diagnostic limitation of evaluating individual biomarkers in isolation. The total pathogenic burden may be underestimated when oxidative or inflammatory markers are measured separately, whereas their combined assessment can more precisely reflect the underlying biological processes that damage sperm [80,81].

Similar to OS, inflammatory signaling within the male reproductive tract likely operates along a biological continuum rather than as a binary pathological state. Low-level or transient inflammatory activity may represent an adaptive or homeostatic response, contributing to tissue surveillance and cellular resilience, whereas sustained or dysregulated inflammation is more likely to exert deleterious effects on spermatogenesis and sperm function. In contrast to ROS, for which hormetic effects and physiological thresholds are relatively well established, comparable dose–response relationships for inflammatory mediators in spermatozoa remain poorly defined. Consequently, current evidence does not support the use of low-level inflammatory markers as standalone indicators of pathology, but rather as contextual signals that should be interpreted alongside OS markers, semen parameters, and clinical findings.

### 5.2. Impact on the Testicular and Seminal Microenvironment

Long-term oxidative and inflammatory signals can especially harm the delicate microenvironment of the testes and the epididymis, which is crucial for healthy spermatogenesis and sperm maturation [7]. Prolonged exposure to inflammatory mediators and ROS alters the function of Leydig and Sertoli cells, thereby impairing germ cell support and dysregulating testosterone synthesis. Concurrently, elevated ROS and cytokine levels in seminal plasma lower TAC, impairing the body’s own antioxidant defense systems and making sperm more vulnerable to oxidative damage [47,58].

Increased sperm DNA fragmentation, decreased motility, and abnormalities in sperm morphology are examples of clinically evident abnormalities that result from these molecular and cellular disruptions [77,82]. The diagnostic significance of assessing oxidative and inflammatory imbalances beyond regular testing is highlighted by the noteworthy fact that such changes can be seen even in individuals with borderline or seemingly normal results in conventional semen analysis [83,84].

### 5.3. Clinical and Diagnostic Implications of the Crosstalk

Male infertility is significantly impacted by the coexistence of OS and inflammation in clinical diseases such as varicocele, genitourinary infections, and systemic metabolic disorders [85]. Concurrent increases in pro-inflammatory cytokines and ROS in semen are frequently observed in patients with various disorders, reflecting a complex clinical condition rather than isolated abnormalities. In both natural conception and ART, this dual burden lowers fertilizing capacity, interferes with embryonic development, and adversely impacts pregnancy outcomes [86,87].

Understanding how oxidative stress and inflammation interact has important therapeutic and diagnostic ramifications. It argues against the fragmented use of individual markers and in favor of comprehensive diagnostic techniques that evaluate both of these pathophysiological pathways concurrently [88,89]. Additionally, identifying combined oxidative–inflammatory characteristics may help classify patients more accurately and direct focused therapy strategies that simultaneously target both pathways. The mechanisms underlying the relationship and its associated clinical implications are summarized in Table 3.

## 6. Biomarkers of OS and Inflammation in Infertility

### 6.1. OS Biomarkers

As a complementary diagnostic approach in the evaluation of male infertility, measurement of OS in sperm and seminal plasma has received increasing attention [102,103]. One of the most researched OS indicators is MDA, a result of lipid peroxidation that indicates oxidative damage to cell membranes. Reduced motility, aberrant sperm shape, and diminished fertilizing capacity have all been repeatedly linked to elevated MDA levels in semen [7]. However, the lack of established reference values and methodological variability in the assays now limit its therapeutic applicability.

Oxidative DNA damage is commonly assessed using 8-OHdG, a marker closely associated with sperm DNA fragmentation and adverse reproductive outcomes [104]. Although 8-OHdG provides valuable mechanistic information on genomic oxidative damage, its application remains largely confined to research and specialized laboratories due to technical complexity and the absence of clinical standardization.

Global measures such as TAC and oxidation-reduction potential (ORP), which reflect the overall balance between oxidative and antioxidant mechanisms in the seminal environment, provide a more comprehensive assessment of redox status [105,106]. By integrating total oxidative and antioxidant activity into a single, reproducible assessment and being supported by commercially available diagnostic tools, ORP has become an attractive diagnostic marker and is now considered one of the most clinically useful methods for evaluating overall oxidative burden in semen [107].

ROS can be measured directly with high sensitivity using techniques such as flow cytometry or chemiluminescence; however, these methods are not yet suitable for routine clinical use due to limited availability, high cost, and technical complexity [108,109]. Overall, despite the substantial diagnostic value of oxidative stress indicators, further validation, standardization, and methodological harmonization are required before widespread clinical implementation. Although universally accepted diagnostic thresholds are lacking, several studies have reported relatively consistent reference ranges for specific OS biomarkers. In particular, ORP has demonstrated reproducible cut-off values across independent cohorts, supporting its potential role as a clinically actionable global redox marker. For example, ORP values above approximately 1.3–1.5 mV/10^6^ sperm/mL have been consistently associated with impaired semen quality in multiple clinical cohorts. These values should be interpreted cautiously and within the context of assay methodology and patient characteristics. To facilitate clinical interpretation, Table 4 summarizes commonly reported reference ranges and proposed cut-off values for selected oxidative stress biomarkers in male infertility, as derived from higher-quality studies cited in this review.

### 6.2. Inflammatory Biomarkers

The relationship between inflammatory indicators in seminal plasma and compromised male reproductive function has been thoroughly investigated [110]. Infertile men frequently exhibit elevated levels of pro-inflammatory cytokines, including IL-6, IL-8, and TNF-α, which have been associated with impaired spermatogenesis and reduced sperm quality [111]. Beyond serving as markers of inflammatory activity, these cytokines act as active mediators that adversely disrupt homeostasis within the seminal tract and testes.

The most readily accessible clinical marker of sperm inflammation is leukocytospermia, defined as the presence of more than 1 × 10^2^ leukocytes per milliliter of semen. However, because it is not consistently linked to male infertility or reproductive outcomes, its diagnostic specificity is low [112]. Enzymatic markers, such as leukocyte myeloperoxidase and elastase, which indicate the level of leukocyte activation and the ensuing oxidative and proteolytic activity, can be used to quantify inflammatory activity more precisely [113,114]. These markers have not been extensively used in clinical practice despite the availability of contemporary immunoassays, primarily because there is disagreement over their true clinical significance and diagnostic criteria.

### 6.3. Diagnostic and Prognostic Relevance

In the treatment of male infertility, the identification of reliable biomarkers of inflammation and oxidative stress has important diagnostic and prognostic implications [12,115]. In cases classified as idiopathic based on conventional sperm analysis, assessment of these biomarkers may help identify underlying oxidative or inflammatory causes, thereby enabling more targeted and rational treatment approaches [14]. For instance, elevated concentrations of cytokines or markers linked to leukocyte activity may indicate a benefit from anti-inflammatory or antimicrobial strategies, whereas elevated levels of MDA or 8-OHdG may indicate a predominant oxidative phenotype and a possible response to antioxidant interventions [116].

Additionally, a number of studies have linked decreased success rates in ART, such as lower rates of fertilization, implantation, and pregnancy attainment, to elevated levels of oxidative and inflammatory biomarkers [117]. However, the strength of these associations varies across studies, reflecting differences in study design, patient selection, biomarker assays, and outcome reporting, which introduce an inherent risk of bias and limit direct comparison of results. Even with these promising results, there is still little incorporation of them into standard clinical practice. The primary obstacles include inter-laboratory variability, the heterogeneity of the approaches employed, the paucity of prospective trials with clinically significant goals, and the lack of well-defined reference values [118].

The creation of standardized procedures and integrated biomarker panels that will concurrently evaluate oxidative and inflammatory pathways is anticipated to be beneficial for future diagnostic techniques. By aligning biomarker evaluation with the principles of precision reproductive medicine, such a combination approach could enhance risk categorization, boost diagnostic accuracy, and enable individualized management of male infertility [119].

Beyond conventional semen parameters, increasing evidence suggests that oxidative stress and inflammatory activity are associated with functional reproductive outcomes, particularly in ART settings. Elevated seminal ROS levels, increased sperm DNA fragmentation, and altered redox balance have been linked to reduced fertilization rates, impaired embryo development, and lower implantation and pregnancy rates in IVF and ICSI cycles. Similarly, inflammatory mediators in seminal plasma have been associated with compromised sperm function and adverse embryological parameters, although their independent contribution remains difficult to quantify due to overlapping oxidative pathways. Importantly, these associations indicate that oxidative–inflammatory biomarkers may provide clinically relevant information regarding sperm fertilizing potential and embryo competence, rather than merely reflecting semen quality, supporting their role as adjunctive diagnostic tools in ART-oriented infertility evaluation.

## 7. Therapeutic Approaches Targeting OS and Inflammation

### 7.1. Antioxidant Supplementation

The administration of antioxidant supplements has been thoroughly investigated as a potential therapeutic option because OS plays a crucial role in the etiology of male infertility. The effects of various vitamins, trace elements, and endogenous antioxidants on sperm parameters and reproductive outcomes have been investigated [120]. For instance, by neutralizing free radicals and preserving the integrity of the cell membrane, vitamins C and E have been linked to increased sperm motility and decreased DNA fragmentation [121]. Similarly, higher sperm quantity and motility have been associated with coenzyme Q10, a crucial component of the mitochondrial respiratory chain, potentially through improved mitochondrial bioenergetic efficiency and reduced oxidative damage [122]. Furthermore, sperm motility and morphology have been positively impacted by L-carnitine and acetyl-L-carnitine, which are essential for the transfer of fatty acids and mitochondrial energy metabolism [123]. Lastly, trace minerals such as zinc and selenium support normal sperm maturation and antioxidant defense, and supplementation with these minerals has been associated with improved sperm quality in certain populations [121].

The aforementioned results highlight the significance of targeted, as opposed to empirical, application of antioxidant treatments from a diagnostic and therapeutic standpoint [124]. The variation in study design, patient selection, baseline levels of OS, as well as the dosage and duration of treatment, is largely responsible for the contradictory clinical results documented in the literature. Antioxidant treatments may be ineffective or potentially harmful if oxidative stress is not assessed beforehand [125]. Consequently, identifying a distinct oxidative phenotype using biomarkers is increasingly recognized as a prerequisite for the rational use of antioxidant therapies. However, establishing optimal and therapeutically evidence-based treatment strategies requires well-designed randomized controlled trials that include baseline oxidative stress assessment and clinically relevant endpoints, such as pregnancy and live birth rates [126].

### 7.2. Anti-Inflammatory Interventions

In the treatment of male infertility, targeting inflammation is another crucial therapeutic strategy. Lifestyle interventions have been associated with improvements in metabolic and reproductive health, as well as reductions in systemic and local reproductive inflammation [70,127]. These interventions include weight loss, regular exercise, quitting smoking, and adopting dietary patterns high in antioxidants, such as the Mediterranean diet. For individuals with inflammation associated with obesity or metabolic problems, these therapies are especially crucial [85].

Targeted pharmacological or surgical therapies may be necessary when inflammation is associated with specific etiological causes. Non-steroidal anti-inflammatory medicines (NSAIDs) can reduce inflammatory activity, but because of possible systemic and reproductive adverse effects, their usage should be done carefully. The preferred course of treatment for inflammation caused by an infectious pathogen is still adequate antibiotic therapy [128]. Simultaneously, varicocele repair is a proven therapeutic technique that can increase sperm parameters and, in certain situations, natural conception rates by lowering testicular oxidative stress and inflammatory signaling [129,130].

Accurate assessment of inflammatory activity using leukocytospermia testing, inflammatory biomarker assays, and clinical evaluation are essential for guiding therapeutic interventions and preventing unnecessary or inappropriate treatment.

### 7.3. Emerging and Future Directions

The transition toward individualized treatment approaches driven by biomarkers that concurrently address inflammation and oxidative stress is increasingly supported by advances in reproductive medicine [131]. A crucial first step toward more accurate and successful management of male infertility is customizing the therapeutic approach based on an individual’s oxidative–inflammatory profile [108]. The use of antioxidant supplements in conjunction with lifestyle changes and/or anti-inflammatory therapy appears to provide synergistic advantages in this setting, enhancing sperm quality more successfully than monotherapy [132].

Novel substances possessing both anti-inflammatory and antioxidant properties, including polyphenols and other bioactive nutrients, are under extensive investigation. Furthermore, pre-treatment approaches that target inflammatory or oxidative conditions prior to the use of ART may improve the quality of gametes and improve clinical outcomes [133].

Future research should prioritize carefully designed clinical trials incorporating biomarker-based patient stratification to enable the integration of these approaches into routine clinical practice. Reporting reproductive outcomes other than sperm characteristics, like conception rates and live birth rates, should be given special attention [134]. Lastly, the establishment of comprehensive diagnostic panels for oxidative–inflammatory activity and the standardization of biomarker assessment are considered crucial for improving clinical efficacy and effectively guiding individualized therapeutic decision making [135].

## 8. Conclusions

Inflammation and OS are closely related processes that are important in male infertility. They adversely impact spermatogenesis and sperm functioning through mechanisms such as lipid peroxidation, DNA damage, mitochondrial failure, and immunological dysregulation. MDA, 8-OHdG, TAC, redox potential, and some inflammatory mediators are examples of indicators of oxidative and inflammatory load that can offer clinically valuable information beyond traditional semen examination, according to accumulated research data. Particularly in situations of idiopathic infertility, these markers help to clarify the underlying etiology. However, due to methodological heterogeneity, a lack of established threshold values, and inadequate prospective clinical validation, their general clinical applicability is still restricted.

The efficacy of current treatment approaches that target oxidative and inflammatory pathways, such as changing one’s lifestyle, taking antioxidant supplements, using anti-inflammatory therapies, and treating underlying pathological conditions like varicocele or infections, often varies, especially when used empirically. Nonetheless, there is growing evidence in support of a shift to a biomarker-driven, individualized strategy, in which the patient’s diagnostic profile informs specific treatment decisions. Future studies should focus on verifying comprehensive diagnostic panels of oxidative and inflammatory activities, as well as standardizing the evaluation of biomarkers. To enable the incorporation of these indicators into the current diagnosis methodology for male infertility and the execution of individualized reproductive treatment, clinical research focused on clinical outcomes is also required.

## Figures and Tables

**Table 1:** This table summarizes the major sources of ROS in the male reproductive system, their mechanisms of action, and the resulting effects on sperm structure, function, and male fertility.

ROS-Generating System/Modulating Condition	Category	Primary Mechanism of ROS Generation	Main Targets of Oxidative Damage	Clinical Consequences on Sperm and Fertility
Immature spermatozoa with residual cytoplasm [5]	Endogenous (cellular)	Increased availability of NADPH substrates supporting enzymatic ROS production (e.g., via oxidoreductase systems)	Plasma membrane lipids, DNA	Reduced motility, abnormal morphology, increased sperm DNA fragmentation
Activated leukocytes (neutrophils, macrophages) [34]	Endogenous/inflammatory	NADPH oxidase–mediated respiratory burst in leukocytes during infection or inflammation (e.g., leukocytospermia)	Lipids, proteins, DNA	Impaired sperm motility, oxidative DNA damage, reduced fertilization potential
Mitochondrial electron transport chain (Complex I and III) [35]	Endogenous (cellular)	Pathological electron leakage from the respiratory chain leading to excessive superoxide generation	Mitochondrial membranes, ATP production	Decreased ATP synthesis, impaired motility, asthenozoospermia
Varicocele [36,37]	Pathological modulator	Testicular hypoxia and venous stasis promoting mitochondrial dysfunction and OS	Lipids, DNA, proteins	Decreased sperm concentration and motility, increased DNA fragmentation
Metabolic disorders (diabetes mellitus, metabolic syndrome, obesity) [38]	Pathological modulator	Chronic metabolic and inflammatory signaling enhancing mitochondrial and NADPH oxidase–dependent ROS generation	DNA, proteins, membranes	Poor sperm quality, reduced fertilization rates, adverse ART outcomes
Cryptorchidism and testicular torsion [39]	Pathological modulator	Ischemia–reperfusion injury inducing mitochondrial ROS overproduction	DNA, membranes	Long-term impairment of spermatogenesis, increased infertility risk
Cigarette smoking [40,41]	Exogenous modulator	Activation of oxidative pathways and depletion of antioxidant defenses, promoting mitochondrial and enzymatic ROS generation	DNA, membrane lipids	Increased DNA fragmentation, reduced motility, abnormal morphology
Alcohol consumption [42]	Exogenous modulator	Acetaldehyde-induced mitochondrial dysfunction and oxidative pathway activation	Lipids, proteins	Reduced sperm concentration and motility
Environmental pollutants (pesticides, heavy metals, air pollution) [43,44,45]	Exogenous modulator	Disruption of mitochondrial function and antioxidant enzymes leading to excess ROS generation	DNA, proteins, membranes	Increased oxidative DNA damage, reduced sperm viability
Poor diet and micronutrient deficiency [46]	Exogenous modulator	Reduced TAC, amplifying the impact of endogenous ROS sources	All sperm cellular components	Increased susceptibility to oxidative damage and idiopathic infertility

**Table 2:** This table summarizes the major inflammatory mediators implicated in male infertility, their primary sources, mechanisms of action, and documented effects on spermatogenesis, sperm quality, and clinical reproductive outcomes.

Inflammatory Mediator/Process	Primary Source	Mechanism of Action	Effects on Spermatogenesis and Sperm Quality	Clinical Associations
IL-6 [58]	Leukocytes, Sertoli cells, seminal plasma	Disruption of Sertoli cell function and steroidogenesis; amplification of inflammatory signaling	Reduced sperm concentration and motility	Genital infections, leukocytospermia, idiopathic infertility
IL-8 [68]	Activated leukocytes, epithelial cells	Chemotactic recruitment of neutrophils and amplification of local inflammatory responses	Increased ROS generation, impaired motility	Prostatitis, epididymitis
TNF-α [15]	Macrophages, leukocytes	Induction of apoptosis, inhibition of spermatogenesis, amplification of OS pathways	Increased sperm DNA fragmentation, abnormal morphology	Chronic inflammation, varicocele
IFN-γ [69,70]	T lymphocytes, immune cells	Disruption of immune privilege and impairment of germ cell support	Impaired sperm maturation and function	Autoimmune-related infertility
Leukocytospermia [6,71]	Activated seminal leukocytes	NADPH oxidase–mediated respiratory burst in leukocytes leading to excessive ROS production	Reduced motility, oxidative DNA damage	Genital tract infections, inflammatory conditions
Varicocele-associated inflammation [72]	Spermatic venous and testicular microenvironment	Increased local inflammatory mediator expression and OS	Decreased sperm quality and testicular dysfunction	Clinical and subclinical varicocele
Systemic inflammatory signaling [73,74,75]	Adipose tissue, metabolic organs	Chronic cytokine release and OS affecting the reproductive tract	Poor sperm quality, reduced fertilization rates	Obesity, metabolic syndrome

**Table 3:** This table depicts the interaction between OS and inflammation in male infertility, highlighting shared mechanisms, effects on the reproductive microenvironment, and clinical implications.

Pathway Interaction	Key Mediators	Mechanism of Crosstalk	Effects on Testicular and Seminal Microenvironment	Clinical and Diagnostic Implications
ROS-induced inflammatory signaling [4,90]	ROS, NF-κB	Activation of redox-sensitive transcription factors leading to cytokine production	Sustained inflammatory signaling and oxidative imbalance	Underestimation of pathology when single biomarkers are assessed
Cytokine-driven ROS amplification [91,92]	TNF-α, IL-6, IL-8	Leukocyte recruitment and activation with increased ROS generation	Reduced antioxidant defenses and increased oxidative damage	Supports combined assessment of OS and inflammation
Oxidative–inflammatory vicious cycle [93,94]	ROS, cytokines, leukocytes	Self-perpetuating feedback loop sustaining tissue injury	Progressive impairment of sperm structure and function	Explains chronic and idiopathic infertility cases
Leydig and Sertoli cell dysfunction [95]	ROS, inflammatory cytokines	Disruption of steroidogenesis and germ cell support	Altered spermatogenesis and reduced testosterone synthesis	Associated with reduced sperm quality despite normal semen parameters
Reduced antioxidant capacity [96]	Elevated ROS and cytokines	Depletion of TAC	Increased susceptibility of sperm to oxidative damage	Justifies TAC measurement as an adjunctive diagnostic tool
Seminal plasma imbalance [97,98]	ROS, cytokines	Altered seminal microenvironment	Increased DNA fragmentation, reduced motility, abnormal morphology	Relevant in ART failure and poor pregnancy outcomes
Disease-associated crosstalk [99,100,101]	Varicocele, infections, metabolic disorders	Concurrent activation of oxidative and inflammatory pathways	Persistent testicular and epididymal dysfunction	Identifies patients who may benefit from combined therapeutic approaches

**Table 4:** Commonly reported reference ranges and proposed cut-off values for selected OS biomarkers in male infertility, as derived from frequently cited clinical studies.

Biomarker	Sample	Commonly Reported Cut-Off/Reference Range	Clinical Association	Key References
MDA	Seminal plasma	Increased compared with fertile controls; commonly >2–3 nmol/mL (method-dependent)	Reduced motility, increased sperm DNA fragmentation	[7,12,29,31]
TAC	Seminal plasma	Reduced in infertile men; often <1.5–2.0 mM Trolox equivalents	Increased susceptibility to oxidative damage	[46,80,96]
ORP	Semen	>1.34–1.48 mV/10^6^ sperm/mL	Global oxidative imbalance; impaired semen quality	[97,105,106,107]
8-OHdG	Sperm DNA	Elevated relative to fertile controls (no universally accepted cut-off)	Increased sperm DNA damage; poor ART outcomes	[26,27,28,104]

## Data Availability

No new data were created or analyzed in this study.

## Abbreviations

The following abbreviations are used in this manuscript:

OS	Oxidative stress
ROS	Reactive oxygen species
IL-6	Interleukin-6
IL-8	Interleukin-8
TNF-a	Tumor necrosis factor-alpha
MDA	Malondialdehyde
8-OHdG	8-hydroxy-2′-deoxyguanosine
ART	Assisted reproductive technology
TAC	Total antioxidant capacity
RPL	Recurrent pregnancy loss
IFN-γ	Interferon–gamma
NF-kB	Nuclear factor kappa B
ORP	Oxidation-reduction potential
NSAIDS	Non-steroidal anti-inflammatory medicines
